# Safety and Effectiveness of Negative Pressure Wound Therapy (Avelle) on Venous Leg Ulcers: The SPACE Study

**DOI:** 10.7759/cureus.95029

**Published:** 2025-10-21

**Authors:** Jorge Ulloa, Valentin Figueroa, Antonio Solano, Gwen Lawrence

**Affiliations:** 1 Vascular Surgery, Fundacion Santa Fe de Bogota, Bogota, COL; 2 Advanced Wound Care, Convatec, Deeside, GBR

**Keywords:** medical device, negative pressure wound therapy, treatment outcome, varicose ulcer, venous leg ulcer

## Abstract

Introduction

Hard-to-heal wounds represent a substantial burden on patients’ quality of life and healthcare systems. Negative pressure wound therapy (NPWT) is an advanced therapy used to manage hard-to-heal wounds as an adjunct to standard wound care; however, there are limited data on its clinical effectiveness. The aim of this study was to evaluate the clinical effectiveness and safety of the Avelle NPWT system in hard-to-heal venous leg ulcers (VLUs).

Methods

This was a single-arm, single-center, open-label, prospective study (NCT05666570) conducted in a Colombian outpatient setting. Patients with a venous ulcer, as per Clinical, Etiological, Anatomical, and Pathophysiological (CEAP) Classification C6/C6R, that has not progressed by >30% in the previous 4 weeks were enrolled. Eligible patients received NPWT at the screening/baseline visit (Visit 1) with scheduled follow-up visits on Day 6 ± 1 (Visit 2) and Day 13 ± 1 (Visit 3; end of study). The primary endpoints were baseline change in wound size and dressing durability. Secondary endpoints included clinical wound characteristics and device-related adverse events.

Results

Fifty-nine patients (median age: 66 years) with VLUs were enrolled (intention-to-treat population). In the per protocol population (n=50), the mean ± standard deviation wound area was 13.4 ± 17.0 cm^2^ at baseline and 7.3 ± 9.9 cm^2^ at study completion (46.8% reduction; p<0.001). Median dressing wear time was 6 days, and changes due to dressing saturation were reported in ≤ 3 (6%) patients. One VLU had completely healed. Erythema was the most common peri-wound skin condition at baseline (n=31, 62%) but decreased at the end of the study (n=26, 54%). Throughout the study, ≤ 6 (13%) patients had signs of eczema/dermatitis, hyperkeratotic callus, maceration, or edema, and there were no signs of infection. One patient experienced device-related dermal lesions resulting in study withdrawal, and one patient had a serious adverse event (not related to the device).

Conclusions

Management of hard-to-heal VLUs with NPWT for 2 weeks was associated with a significant reduction in wound size, as well as effective exudate control and a good safety profile. These results, combined with features of the device such as portability and ease of use, suggest it could be an effective treatment option in hard-to-heal wounds.

## Introduction

Despite therapeutic advances in wound care, the number of hard-to-heal wounds is increasing [1-4]. Both acute and chronic wounds can be hard-to-heal, independent of wound type and etiology [4-6]. The term ‘hard-to-heal’ has been increasingly favored instead of “chronic” to signify that wounds are not lifelong and that the barriers to healing (i.e., biofilm) can be overcome [5,6]. The global prevalence is estimated to be ~1.9 per 1000, and it is predicted that the incidence will rise with the aging population [1-3]. Hard-to-heal wounds are associated with a significant reduction in health-related quality-of-life (QoL) and are a major burden on healthcare systems [3,7-10]. Venous leg ulcers (VLUs), diabetic foot ulcers (DFUs), and pressure ulcers are wounds that are frequently reported as hard-to-heal [4]. The risk of developing hard-to-heal wounds increases with patient-related factors such as age, underlying pathology, comorbidities (e.g., malnutrition and obesity), and wound-related factors such as duration, size, wound bed condition, presence of ischemia or infection/inflammation, anatomical site, and response to treatment [4,11]. Management of hard-to-heal (or chronic) wounds aims to promote healing, control infection, reduce pain, and improve patient QoL [4,12]. The skills and knowledge of healthcare professionals are an increasingly important factor in providing best practice care due to the complexity of such wounds and the multitude of available dressings and advanced therapies [11-14].

While compression therapy in combination with modern dressings remains the mainstream management option for VLUs, in selective cases (e.g., highly exuding wounds), other approaches may be helpful. Negative pressure wound therapy (NPWT) is an advanced therapy used to manage hard-to-heal wounds as an adjunct to standard wound care [4,15]. NPWT systems apply sub-atmospheric pressure to continuously remove wound exudate and small tissue debris and pull together wound edges, ultimately reducing wound size [12,16]. The sealed system also promotes scar tissue formation by maintaining a moist wound environment and prevents the entry of external bacteria [16]. Potential benefits of single-use NPWT in hard-to-heal wounds versus standard dressings include earlier hospital discharge, reduction in required dressing changes and associated costs, and improved patient QoL [16-18]. NPWT is indicated for use on selected patients who would benefit from NPWT devices on wounds with low-to-moderate exudate, including hard-to-heal, acute, subacute, and dehisced, and traumatic wounds, flaps, and grafts, and surgically closed incision sites [19-21]. There is limited data on the clinical effectiveness of NPWT on hard-to-heal wounds. Cochrane systematic reviews published in 2015-2018 concluded that there was low-level evidence for the benefit of NPWT versus standard care for VLUs, DFUs, and pressure ulcers [22-24]. For VLUs specifically, clinical guidelines from the Society for Vascular Surgery, American Venous Forum, and European Society for Vascular Surgery do not recommend the routine primary use of NPWT for management of VLUs due to lack of evidence [25,26]. The objective of the SPACE study was to address the evidence gap by evaluating the safety and effectiveness of the Avelle NPWT system in hard-to-heal VLUs.

## Materials and methods

Study design

The SPACE study is a single-arm, single-center, open-label, prospective study (NCT05666570) designed to evaluate the safety and effectiveness of NPWT (Avelle; Convatec Ltd., Deeside, UK) on hard-to-heal wounds. The study was conducted in an outpatient setting in Colombia between May and October 2022. Eligible patients received NPWT at the screening/baseline visit (Visit 1) with scheduled follow-up visits on Day 6 ± 1 (Visit 2) and Day 13 ± 1 (Visit 3; end of study). Unscheduled visits were allowed at the discretion of the investigator and dependent on wound status. Patients received NPWT until study completion or withdrawal. At Visit 1, patient eligibility was assessed, baseline characteristics were collected, and the wound area was evaluated prior to NPWT system initiation. At Visits 2 and 3, dressings were changed, wound area evaluated, and adverse events recorded (if any).

Study oversight and review

The study was conducted in accordance with the ICH GCP and the Declaration of Helsinki. The study protocol and informed consent form were reviewed and approved by the Convatec Wound Clinical for Institutional Review Board (IRB) in Colombia. Safety was monitored by Convatec Safety & Compliance. All patients provided written informed consent before placement of the device.

Objectives and endpoints

This study aimed to evaluate the safety and effectiveness of NPWT (Avelle) on hard-to-heal wounds when used in accordance with its Instructions for Use (IFU). The primary endpoints were the change in wound area from baseline (Visit 1) to the end of the study (Visit 3) and dressing durability as defined by time (days) to dressing strikethrough (loss of edge seal, adherence, leaks, or dislodgement) and the number of dressing changes. Wound area was determined by measuring the length and width of the VLU using a ruler. Secondary endpoints were clinical wound characteristics (including wound healing status, peri-wound skin condition, and signs of infection) and device-related adverse events (AEs).

Study device and instructions for use

Avelle is a disposable NPWT system comprising a battery-powered pump unit and a range of absorbent dressings connected by flexible airway tubing. The system delivers a negative pressure level of 80 mmHg (± 20 mmHg) to remove exudate from the wound surface area in conjunction with the absorbent dressing. The dressing is composed of a perforated silicone adhesive border, layers of sodium carboxymethylcellulose fabric that gels on contact with wound fluid (Hydrofiber Technology), a polyester foam layer to aid distribution of negative pressure, and a protective polyurethane cover layer that allows exudate evaporation. Three dressing sizes were used in the study (16×16 cm, 16×21 cm, and 21×26 cm). The Avelle NPWT system is indicated for use in patients who would benefit from a NPWT device with a low-to-moderately exuding wound, such as chronic wounds, e.g., leg ulcers. The pump unit has a maximum usage time of 30 days, and dressings have a maximum wear time of 7 days.

Study participants

Patients included in the study had a clinical history of chronic venous insufficiency documented by duplex ultrasound and/or supported by physical examination. The full inclusion criteria for enrollment were as follows: ≥ 18 years of age; able and willing to provide informed consent; venous ulcer (Clinical, Etiological, Anatomical, and Pathophysiological (CEAP) Classification C6 or C6R); stalled wound/failed treatment in the opinion of the investigator (wound that has not progressed by > 30% in the previous 4 weeks); one qualifying wound amenable to NPWT with low-to-moderate exudate; and able to tolerate negative pressure. Exclusion criteria included: wounds greater than 12 months duration; previous NPWT failure within last 6 weeks on the qualifying wound; known sensitivities or allergies to components of the study device; necrotic wounds or eschar present; wound area that too small or large for dressing, wound depth > 2 cm; use of hydrocolloid gel (DuoDERM) or petroleum/oil-based gels or creams; treatment for cancer ≤ 3 months expected survival; severe malnutrition; visible bone/ tendon or exposed articular capsule; exposed blood vessels; clotting disorder; malignant wounds; systemic infection; untreated osteomyelitis; active pregnancy; and Chronic Kidney Disease stage 5.

Statistical analyses

The null hypothesis was that management of hard-to-heal wounds with NPWT does not impact wound progression or management of wound exudate. Paired t-tests and non-parametric tests were used to detect a significant change in wound size from baseline, with a P value of < 0.05 considered statistically significant. Safety endpoints (AEs) are reported for the intention-to-treat (ITT) population (N = 59), comprising all eligible patients who enrolled in the study. Efficacy endpoints (wound size, dressing durability, and clinical wound characteristics) are reported for the per protocol (PP) population (n = 50), which comprised patients who received the study device as per the IFU.

## Results

Patients

In total, 59 patients with VLUs were enrolled during the study period (18 May 2022 to 12 October 2022; ITT population). One patient had discontinued NPWT at Visit 2 due to an adverse event. Fifty-nine NPWT pump units were used with 119 dressings of three sizes: 16×16 cm (42 (35%)), 16×21 cm (58 (49%)), and 21×26 cm (19 (16%)). Fifty-four patients received compression therapy (double-layer bandage) at baseline (Visit 1), and 52 received compression therapy at Visit 2. The median age was 66 years (range, 31-82), 41 (69%) patients were female, and all patients were Hispanic or Latino (Table 1). Median body mass index was 27.7 (range: 21.5-40.8), and the most common comorbidities were hypertension (23 (39%)) and thyroid disease (14 (24%)). All wounds were VLUs of varying age: 1-3 months (15 (25%)), 3-6 months (21 (36%)), 6-9 months (10 (17%)), and 9-12 months (13 (22%)). The wounds were located in the ankle (22 (37%)), calf (19 (32%)), or gaiter (18 (31%)), and most were positioned medially (36 (61%)). Baseline characteristics are summarized in Table 1.

**Table 1:** Baseline characteristics *All eligible patients who enrolled in the study. ^†^Patients who received the study device as per the IFU. ^‡^Only comorbidities with frequency ≥3 are reported. BMI: body mass index; IFU: Instructions for Use; ITT: intention-to-treat; PP: per protocol

Characteristic		ITT Population^*^ (N = 59)	PP Population^†^ (n = 50)
Age, median (range)		66 (31–82)	67 (31–82)
Gender, n (%)	Male	18 (31)	16 (32)
	Female	41 (69)	34 (68)
BMI, median (range)		27.7 (21.5–40.8)	27.7 (21.5–40.8)
Comorbidities, n (%)^‡^	Hypertension	23 (39)	20 (40)
	Thyroid disease	14 (24)	12 (24)
	Other cardiovascular disorders	12 (20)	10 (20)
	Lipid disorder	5 (8)	3 (6)
	Diabetes mellitus	4 (7)	3 (6)
	Hypercholesterolemia	4 (7)	4 (8)
	Medication allergy	3 (5)	3 (6)
	Chronic musculoskeletal pain	3 (5)	1 (2)
	COPD	3 (5)	3 (3)
Wound duration, n (%)	> 1 to < 3 months	15 (25)	10 (20)
	> 3 month to < 6 months	21 (36)	19 (38)
	> 6 month to < 9 months	10 (17)	10 (20)
	> 9 months to < 12 months	13 (22)	11 (22)
Wound location, n (%)	Left	35 (59)	29 (58)
	Right	24 (41)	21 (42)
Wound position, n (%)	Anterior	3 (5)	3 (6)
	Lateral	20 (34)	17 (34)
	Medial	36 (61)	30 (60)
Wound anatomical location, n (%)	Ankle	22 (37)	19 (38)
	Calf	19 (32)	15 (30)
	Gaiter	18 (31)	16 (32)

Wound size

**Figure 1:**
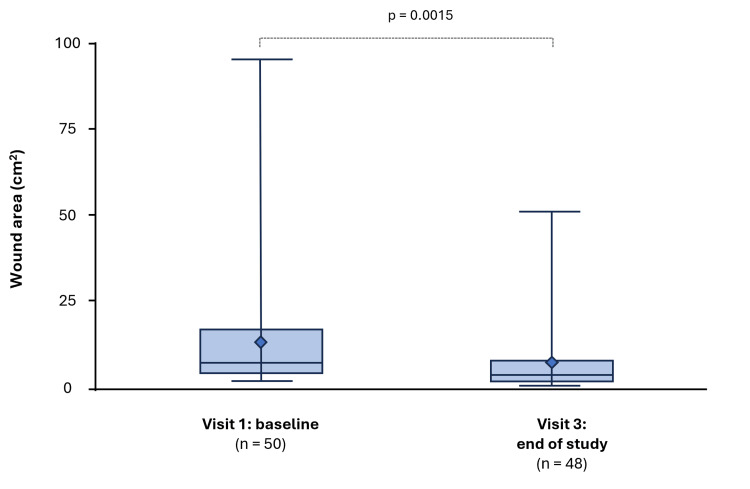
Boxplot of wound area over the study period (PP population) PP: per protocol

In the PP population (n = 50), the mean ± standard deviation (SD) wound area was 13.4 ± 17.0 cm^2^ at baseline and 7.3 ± 9.9 cm^2^ at the end of the study (Visit 3) (Figure 1); this corresponded to a statistically significant mean reduction of 6.3 ± 12.9 cm^2^ (p<0.05). The mean (SD) percentage reduction in wound area from baseline to end of study was 46.8 ± 24.3% (p<0.001) (Table 2).

**Table 2:** Wound size by study visit and change at Visit 3 (PP population) *Day 13 ± 1. Paired t-tests and non-parametric tests were used to detect a significant change in wound size from baseline, with a P value of < 0.05 considered statistically significant. CI: confidence interval; PP: per protocol; SD: standard deviation

	Visit 1: Baseline (n = 50)		Visit 3: End of Study* (n = 48)		Absolute Change (n = 48)					Percentage Change (n = 48)			
	Mean (SD)	Median (range)	Mean (SD)	Median (range)	Mean (SD)	95% CI	Median (range)	T value	P value	Mean (SD)	95% CI	Median (range)	P value
Area (cm^2^)	13.4 (17.0)	7.1 (1.9–95.0)	7.3 (9.9)	3.6 (0.4–51.0)	6.3 (12.9)	2.5–10.0	3.3 (-0.3–88.0)	3.38	0.0015	46.8 (24.3)	39.7–53.8	48.1 (-3.3–92.6)	<0.001
Length (cm)	4.2 (2.3)	3.40 (1.0–1.0)	3.2 (2.1)	2.5 (0.8–9.3)	1.0 (1.1)	0.7–1.4	0.8 (0.0–6.5)	6.42	<0.0001	25.4 (18.0)	20.1–30.6	23.7 (0.0–66.0)	<0.0001
Width (cm)	2.6 (1.7)	2.4 (1.0–9.5)	1.8 (1.3)	1.5 (0.5–6.0)	0.9 (1.1)	0.5–1.2	0.5 (-0.1–7.5)	5.21	<0.0001	31.4 (21.7)	25.1–37.7	33.3 (-3.3–79.2)	<0.0001

Dressing durability

Dressings were changed at the scheduled visits per protocol. The median wear time at both study visits was 6 days (Table 3). Changes due to dressing saturation were reported in ≤ 3 (6%) patients at Visits 2 and 3. Dressing changes for wound inspection were reported in 1 (2%) and 15 (31%) patients at Visit 2 and Visit 3, respectively. There was one (2%) change due to dressing lift at Visit 3.

**Table 3:** Dressing wear time and reasons for dressing change (PP population) *Day 6 ± 1. ^†^Day 13 ± 1. One dressing change was due to a wound inspection during an unscheduled visit. IFU: Instructions for Use; PP: per protocol; SD: standard deviation

	Visit 2: follow-up* (n = 49)	Visit 3: end of study^†^ (n = 48)
Dressing wear time since the previous visit, days		
Mean (SD)	5.8 (0.9)	5.7 (1.4)
Median (range)	6.0 (3.0–7.0)	6.0 (2.0–7.0)
Reason for dressing change, n (%)		
Wound inspection	1 (2)	15 (31)
Dressing saturation per IFU	3 (6)	1 (2)
Dressing lift	0	1 (2)

Clinical wound characteristics

**Figure 2:**
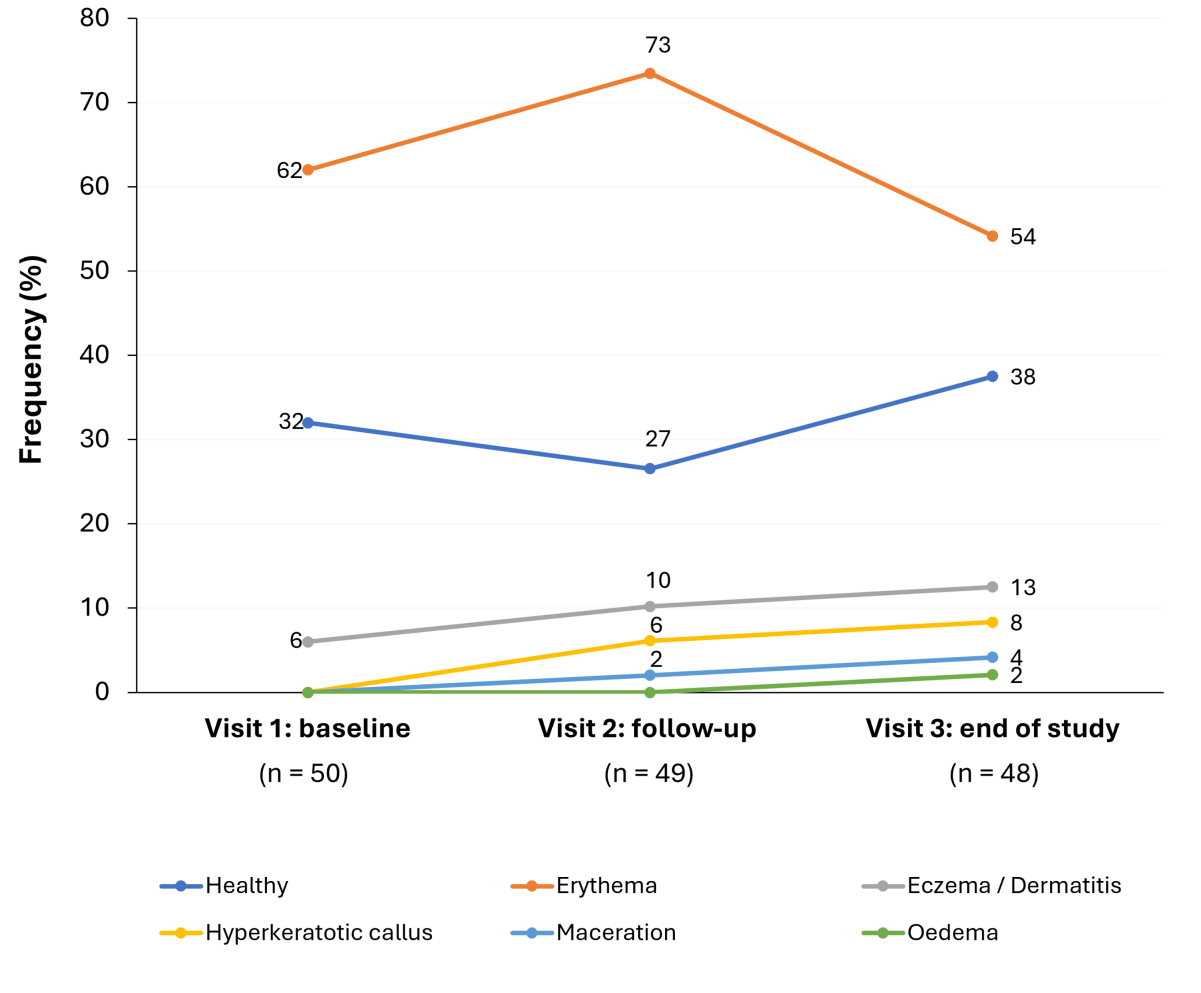
Peri-wound skin condition by Visit (PP population) Two peri-wounds were evaluated as healthy during unscheduled visits. Numbers may not total to 100 due to rounding. PP: per protocol

**Figure 3:**
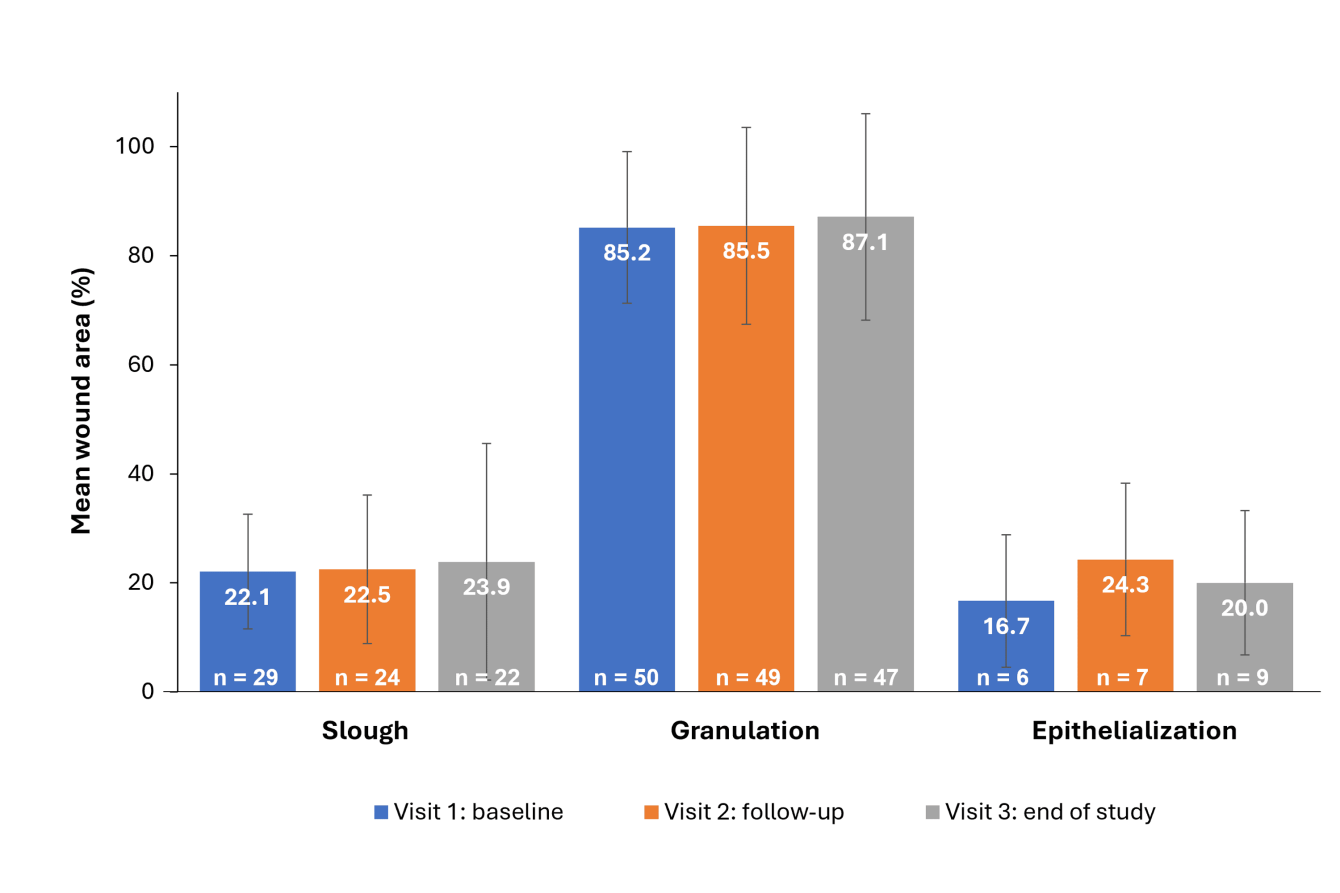
Mean percentage of slough, granulation, and epithelial tissue in wound (PP population) Error bars represent standard deviation. PP: per protocol

One patient’s VLU had healed by the end of the study (Visit 3). Throughout the study, ≤ 6 (13%) patients had signs of eczema/dermatitis, hyperkeratotic callus, maceration, or edema in the peri-wound skin (Figure 2). Erythema was the most common peri-wound skin condition at baseline (31 (62%)) but decreased at the end of the study (26 (54%)). Surface skin temperatures were normal for all patients at all visits. The percentage of slough, granulation, and epithelial tissue reported in wounds marginally increased from baseline to the end of the visit (Figure 3).

Adverse events

One patient experienced an AE related to the device at Visit 2. The patient developed additional dermal lesions that merged with the initial VLU around the silicone contact area of the dressing, leading to increased exudate and pain. The patient was withdrawn from the study, and the AE resolved. Additionally, one patient experienced a serious AE that was not considered device-related. This patient attended the emergency room following 8 days of pain in the left lower limb and was hospitalized. The diagnosis was varicose veins of the lower limb with ulceration and inflammation, and resolved.

## Discussion

VLUs are the most common hard-to-heal wounds and represent a substantial burden on patient QoL and healthcare costs [7,27]. In this single-arm, single-center, open-label, prospective study, we evaluated the clinical effectiveness and safety of an NPWT device in hard-to-heal VLUs. We defined a hard-to-heal wound in this study as one that has not progressed by > 30% in the previous 4 weeks; however, there is currently a lack of consensus on this definition [28]. Nevertheless, Martin & Nunan defined a “chronic wound” as a barrier defect that has not healed in 3 months, and Leaper & Durani defined it as a wound that lacked a 20-40% reduction in size after 2-4 weeks of optimal treatment or when there is incomplete healing after 6 weeks [28,29].

Management of hard-to-heal VLUs with NPWT (Avelle) was associated with a significant reduction in wound area after two weeks (46.8%), including complete healing in one patient. Moreover, NPWT was associated with a good safety profile, with only one AE related to the device. The attrition rate was 1.7% (one patient) during the study period, suggesting that the NPWT device was well tolerated. Most dressings were changed to evaluate the wound area, and ≤ 3 (6%) patients had dressing changes due to saturation, highlighting effective exudate management. There were no notable changes in peri-wound skin condition, although the most common baseline condition (erythema) decreased by the end of the study. Moreover, there were no signs of infection/inflammation throughout the study, which could be attributed to the sealed system protecting against external microorganisms.

While cross-trial comparisons are difficult to make due to differences in study design and populations, our results are consistent with previously published studies of NPWT in hard-to-heal wounds. A prospective, randomized, controlled study, which included 53 patients with VLUs receiving NPWT or routine dressings for 2 weeks, reported significantly higher healing rates with NPWT [30]. Additionally, a prospective cohort study including 326 patients with wounds of mixed etiology (28% with VLUs, DFU, or pressure ulcers) receiving NPWT reported a median wound area reduction of 35% from baseline at week 3, 62% at week 6, and 78% at week 8 [31]. Our findings support the limited available data on the clinical effectiveness of NPWT in the hard-to-heal VLU population.

Our study has several noteworthy limitations. Firstly, the study was conducted in a single site in Colombia, comprising Hispanic or Latino patients only, and therefore, the applicability of our findings to the general population is unclear. Secondly, the single-arm nature of the study makes it difficult to distinguish between the impact of NPWT versus the natural healing trajectory of the wound; however, our study included patients with chronic wounds that had failed standard therapies. Thirdly, the included wounds may be considered small (mean 13.4 cm^2^) and not representative of all VLUs. However, studies have reported that an ulcer area greater than 10 cm^2^ is a significant predictor of delayed healing [32]. Lastly, the duration of this study was short, and it is unknown if more patients would achieve complete healing beyond 2 weeks of NPWT. Future directions of our research could include longer treatment/follow-up time, as well as analyses to identify subgroups who may better benefit from NPWT.

## Conclusions

In conclusion, this prospective, single-arm study contributes to the growing body of evidence supporting the use of NPWT in the management of hard-to-heal VLUs, demonstrating a meaningful reduction in wound area and a favorable safety profile over a two-week treatment period. The significant wound size reduction, coupled with minimal AEs, highlights the potential of NPWT as an effective therapeutic option for hard-to-heal wounds unresponsive to standard care. The device was well-tolerated, as reflected by the low attrition rate and efficient exudate management, with few dressing changes required due to saturation. While the study’s limitations, including its single-center design, short follow-up duration, and limited patient diversity, restrict the generalizability of the findings, they also underscore important directions for future research. Overall, our findings offer valuable insights into the role of NPWT in facilitating healing in a challenging patient population. Combined with its portability and ease of use, the Avelle system may represent a promising and practical treatment option for managing complex, hard-to-heal VLUs in real-world clinical settings.
